# Robotic Stereotactic Assistance (ROSA) for Pediatric Epilepsy: A Single-Center Experience of 23 Consecutive Cases

**DOI:** 10.3390/children7080094

**Published:** 2020-08-07

**Authors:** Jonathon H. Nelson, Samantha L. Brackett, Chima O. Oluigbo, Srijaya K. Reddy

**Affiliations:** 1 Division of Anesthesiology, Pain and Perioperative Medicine, Children’s National Hospital, The George Washington University School of Medicine & Health Sciences, Washington, DC 20010, USA; sbrackett@childrensnational.org; 2 Division of Neurosurgery, Children’s National Hospital, The George Washington University School of Medicine & Health Sciences, Washington, DC 20010, USA; coluigbo@childrensnational.org; 3 Department of Anesthesiology, Division of Pediatric Anesthesiology, Monroe Carell Jr. Children’s Hospital, Vanderbilt University Medical Center, Nashville, TN 37232, USA; srijaya.k.reddy@vumc.org

**Keywords:** refractory epilepsy, robotic surgical procedures, pediatric neurosurgery, perioperative care, pediatric anesthesia

## Abstract

Robotic assisted neurosurgery has become increasingly utilized for its high degree of precision and minimally invasive approach. Robotic stereotactic assistance (ROSA^®^) for neurosurgery has been infrequently reported in the pediatric population. The goal of this case series was to describe the clinical experience, anesthetic and operative management, and treatment outcomes for pediatric patients with intractable epilepsy undergoing ROSA^®^ neurosurgery at a single-center institution. Patients who underwent implantation of stereoelectroencephalography (SEEG) leads for intractable epilepsy with ROSA^®^ were retrospectively evaluated between August 2016 and June 2018. Demographics, perioperative management details, complications, and preliminary seizure outcomes after resective or ablative surgery were reviewed. Nineteen children who underwent 23 ROSA^®^ procedures for SEEG implantation were included in the study. Mean operative time was 148 min. Eleven patients had subsequent resective or ablative surgery, and ROSA^®^ was used to assist with laser probe insertion in five patients for seizure foci ablation. In total, 148 SEEG electrodes were placed without any perioperative complications. ROSA^®^ is minimally invasive, provides superior accuracy for electrode placement, and requires less time than traditional surgical approaches for brain mapping. This emerging technology may improve the perioperative outcomes for pediatric patients with intractable epilepsy since large craniotomies are avoided; however, long-term follow-up studies are needed.

## 1. Introduction

**Figure 1:**
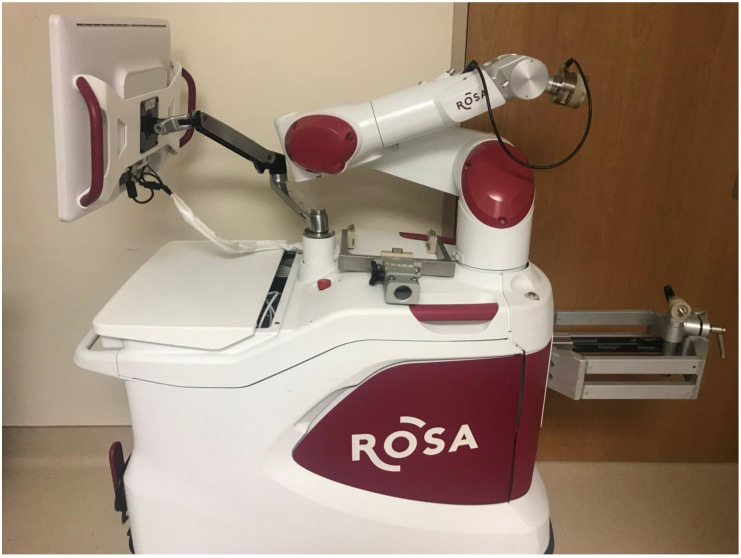
Robotic stereotactic assistance (ROSA^®^) robot.

**Figure 2:**
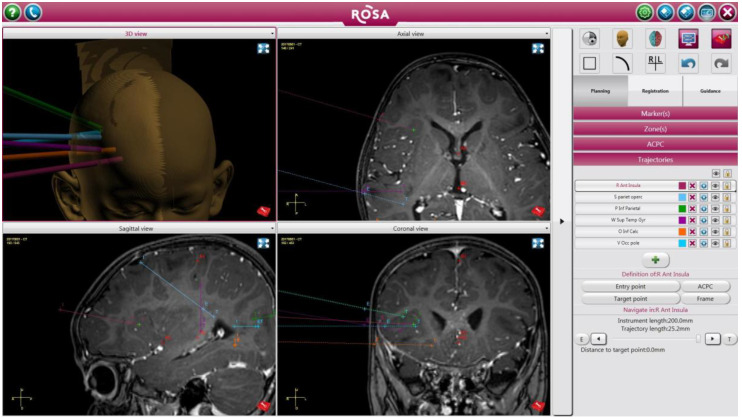
Planned depth electrode trajectories based on the patient’s imaging studies uploaded on the ROSA^®^ system.

Robotic stereotactic assistance, or ROSA^®^ (Medtech, Montpellier, France), is a computer-controlled robotic arm with an integrated platform that combines image-guided neurosurgical planning software with robotic navigation to assist neurosurgeons with minimally invasive procedures. ROSA^®^ incorporates four main components: the robot stand, a retractable telescopic support arm, a touch-screen, and a robotic arm (Figure 1). Preoperative images are registered to the patient’s facial features via laser scanning; ROSA^®^ then aligns to the appropriate planned trajectory (Figure 2). The system allows for planning of multiple trajectories using computerized tomography (CT) and magnetic resonance imaging (MRI) [1]. ROSA^®^ has been used for functional and stereotactic neurosurgery, endoscopic surgery, and open skull procedures. Its use is most reported for lead placement for deep brain stimulation, frameless stereotactic biopsies, laser ablation of epileptogenic foci, endoscopic third ventriculostomy, and depth electrode placement for seizure monitoring [1,2,3,4].

ROSA^®^ is of particular interest in the pediatric population, as many brain pathologies in children present a considerable neurosurgical challenge. Normal brain anatomy can be compromised, and a child’s developing brain is extremely vulnerable to injury [2]. Therefore, an accurate image-guided and minimally invasive approach to many pediatric neurosurgical procedures is highly desirable.

While applications for ROSA^®^ are expanding in the adult population, the utility in pediatric neurosurgery has been infrequently reported [1]. Furthermore, the anesthetic considerations for pediatric ROSA^®^ procedures remain to be discussed. In this case series, we present our experience with 19 pediatric patients with intractable epilepsy undergoing 23 ROSA^®^ procedures for stereoelectroencephalography (SEEG) depth electrode placement.

## 2. Materials and Methods

This study was approved by the Children’s National Hospital Institutional Review Board on 6/10/2019 with a need for consent waiver. All pediatric patients with a primary diagnosis of epilepsy who underwent ROSA^®^-assisted procedures for management of intractable epilepsy from August 2016 to June 2018 were retrospectively evaluated. Electronic medical records were reviewed for demographic and surgical data, anesthetic management techniques, and postoperative outcomes. Numbers and percentages were determined for all categorical variables. Mean ± standard deviation and median with range were calculated for continuous variables.

## 3. Results

Twenty-three ROSA^®^ procedures were performed in 19 patients. Demographics and outcomes are displayed in Table 1 and Table 2. ROSA^®^-assisted SEEG implantation of 148 total electrodes occurred without complications. No patients experienced hemorrhage after electrode placement, as confirmed by either postoperative CT scan or intraoperative MRI (iMRI). The iMRI was used in conjunction with Visualase^®^ (Medtronic, Minneapolis, MN, USA) seizure foci ablation in five patients. There was no incidence of surgical site infection, nor did any patients require electrode re-implantations; mean operative time was 148 min. The average hospital length of stay needed for seizure localization was 9 days, with one patient requiring a 29-day hospital stay due to difficulty with seizure localization and refractory epilepsy (Table 1).

### 3.1. Anesthetic Management

#### 3.1.1. Preoperative Evaluation, Induction, and Maintenance

Patients underwent routine preoperative workup including labs for complete blood count, basic metabolic panel, and coagulation panel. Benzodiazepines for premedication were avoided due to potential interference with intraoperative electrocorticography (ECoG). Background ECoG represents basal cortical activity without interference from the scalp and skull, although signals vary with electrode location, pre-existing brain lesions, preoperative medications, and anesthetics. Patients underwent general anesthesia with standard monitoring and were either induced via mask with 8% sevoflurane and 70% nitrous, followed by peripheral intravenous (IV) catheter insertion, or by IV induction using 2% lidocaine 1 mg/kg and propofol 2–3 mg/kg (Table 1). Following induction of anesthesia, most patients were administered fentanyl 0.5–1 µg/kg and rocuronium 0.5 mg/kg and endotracheally intubated. At least two large-bore IV catheters were inserted in the rare chance of acute blood loss or hemodynamic instability. A radial arterial line was place in 30% of patients (Table 1).

General anesthesia was maintained with sevoflurane or isoflurane at 0.5 minimum alveolar concentration (MAC). Most patients were maintained on an IV infusion of remifentanil 0.1–1 µg/kg/min. Nine patients were not maintained on a continuous opioid infusion, receiving fentanyl and/or morphine boluses instead. Patients also received IV acetaminophen and up to 4 µg/kg of fentanyl in divided doses. Rocuronium was used in most cases to ensure no patient movement occurred; however, for five patients, paralytic was not administered due to motor evoked potentials monitoring (Table 1). All patients received antibiotics to prevent surgical site infection, and dexamethasone and ondansetron to prevent postoperative nausea and vomiting.

Prior to ECoG, sevoflurane was discontinued, and isoflurane was used to reduce interference with ECoG. In three cases, isoflurane was used throughout the entire surgery (Table 1). During ECoG recording, isoflurane was minimized to 0.25 MAC and used with 50% nitrous oxide along with either the continuous opioid infusion or opioid boluses.

#### 3.1.2. Positioning and Protection

All patients were positioned supine with arms padded and tucked. Sterile lubricant eye ointment was used prior to taping the eyes to prevent corneal injury. All patients had a Foley catheter. Forced-air warming blankets were used to prevent hypothermia, and sequential compression devices were used for patients >40 kg to prevent deep vein thrombosis. The operating room table was rotated 90° away from the anesthesia machine, and the patient’s head was pinned in a Mayfield^®^ frame (Integra, Plainsboro, NJ, USA) and then locked to the ROSA^®^ telescopic arm (Figure 1). Once optimal positioning was confirmed, the handheld bed control was disconnected from the operating room table, and the table was unplugged to prevent inadvertent movement. This technique for patient positioning during the ROSA^®^ procedure was utilized to ensure the best surgical exposure with ample space for the ROSA^®^ robot and surgical equipment.

#### 3.1.3. Hemodynamic Control and Ventilation Strategies

Mean arterial blood pressure was maintained within 20% of baseline pressures. Hypertension was managed by increasing the rate of narcotic continuous infusion and/or with opioid boluses. Hypotension was treated with crystalloid and/or 5% albumin boluses, by titrating down the rate of narcotic infusion or with ephedrine for persistent hypotension during ECoG. Seven patients required albumin, while five patients required ephedrine. No patients required a continuous infusion of a vasoactive. No patients required a blood transfusion; however, one patient received a 4.4 mL/kg platelet transfusion empirically per the surgeon’s request. All patients were placed on controlled mechanical ventilation to reduce the chance of coughing or movement.

#### 3.1.4. Emergence and Postoperative Care

After ensuring that all depth electrodes were functional, the patient’s head was removed from the pins and frame, and an awake extubation was performed in order to facilitate immediate neurological assessment. Postoperative pain was typically controlled with IV acetaminophen every six hours and with opioids for breakthrough pain. No patients experienced any anesthetic complications or had issues with inadequate pain control.

### 3.2. Surgical Management

The patient’s head was held in three-point head pin fixation using Mayfield^®^ or Leksell^®^ (Elekta, Stockholm, Sweden) head frame secured with pins and clamps using a pressure of 40 pounds. The Mayfield^®^ clamp was then connected to the ROSA^®^ neurosurgical robot support telescopic arm as well as the Mayfield^®^ adapter on the surgical bed (Figure 1). The patient was then registered to the previously acquired brain MRI and CT images using laser scan of the forehead and facial landmarks identified with a distance calibrator. Once the accuracy was verified, the ROSA^®^ stereotactic neuro-navigation software planned the trajectories (Figure 2). In this plan, entry points (EP) and target points (TP) were identified using multiplanar reconstructions of the brain while avoiding all vascular structures. Following completion of the trajectory planning, the head was prepped and draped in a sterile fashion.

The ROSA^®^ was then aligned with the instrument holder at the EP of each planned trajectory and an incision was made at this point. A drill was utilized to make a burr hole, and once the dura was opened, a bone anchor was affixed to the skull. The distance from the top of the bone anchor to the TP was calculated. Subsequently, an SEEG depth electrode of standard diameter (1.1 mm) was then measured to the TP from the top of the robot instrument holder. The SEEG depth electrode was then introduced into the TP along this trajectory to the appropriate depth, and the locking brackets were applied to fix the electrode. Upon placement of all electrodes, an intraoperative SEEG depth ECoG was performed to ensure that accurate electrocorticographic signals were obtained prior to extubation. During the study period, all of the leads were appropriately placed for seizure localization, and no leads needed to be repositioned or replaced.

All patients remained in the pediatric intensive care unit (PICU) overnight for frequent neurologic evaluation and were transferred to the neurosurgical floor the next day for close monitoring of seizure activity and localization via the depth electrodes. A postoperative head CT was obtained for all patients except for those who had an iMRI as part of their surgery, to rule out an intracranial bleed, and intraoperative estimated blood loss was minimal (<20 mL) in each case (Table 2). There were no intraoperative surgical complications or postoperative evidence of intracranial bleed or neurological deficits.

### 3.3. Illustrative Case: Patient 10

#### 3.3.1. Preoperative Evaluation

A 12-year-old 58 kg female with refractory focal epilepsy secondary to right frontal cortical dysplasia presented for surgery. She previously had three craniotomies to remove areas of cortical dysplasia. Despite prior surgical intervention, she continued to have increased seizure frequency.

#### 3.3.2. Anesthetic Management

She was induced via a mask with 8% sevoflurane and 70% nitrous oxide. Upon placement of a 20-gauge IV, nitrous oxide was discontinued, and propofol 2 mg/kg and fentanyl 0.5 µg/kg were administered. She was intubated with a 6.5 cuffed endotracheal tube, and an additional 20-gauge IV was placed. Sevoflurane was reduced to 1.2% with a mixture of air and oxygen at 50% FiO_2_, and a remifentanil 0.2 µg/kg/min continuous IV infusion was initiated. The bed was then turned 90° toward the ROSA^®^ robot. She received an additional 1 mg/kg propofol bolus prior to the insertion of Mayfield^®^ pins. She was appropriately positioned and padded and administered cefazolin 30 mg/kg prior to incision. Prior to the ECoG, sevoflurane was switched to 0.5% isoflurane and 50% nitrous oxide was added. After ECoG completion, nitrous was discontinued. She received a total of 30 mL/kg of crystalloid, fentanyl 2 µg/kg, ondansetron 4 mg, dexamethasone 3 mg, and IV acetaminophen 15 mg/kg. She was extubated awake at the end of surgery and transferred to PICU for further monitoring.

#### 3.3.3. Operative Details

A total of four SEEG electrodes were placed via four burr holes without any complications or postoperative neurologic deficits. ECoG recordings were optimal during the procedure, which helped minimize operative time to 67 min (Table 2).

#### 3.3.4. Postoperative Course

She was monitored overnight in the PICU and transferred to the floor for the next 10 days, where she was monitored for seizure activity via SEEG. Following removal of the SEEG electrodes under general anesthesia on postoperative day 10, she was discharged home. Once SEEG data were reviewed, it was determined that she was a candidate for a Visualase^®^ procedure, which was performed eight weeks later. Since her Visualase^®^ procedure, she has a decreased seizure frequency and intensity, with improved performance in school. She continues to be followed to evaluate her disease progression.

## 4. Discussion

Robotic implantation of SEEG electrodes has many advantages over other frame-based methods of neuro-navigation including optimization of diagnostic accuracy, particularly given that stereotactic target accuracy is inversely related to trajectory length, which is significant when reaching deeper brain targets during SEEG implantation [5,6]. Furthermore, robotic assistance allows several trajectory options without requiring numerous frame adjustments, shortening operative duration and reducing potential complications [7]. Because of the need for accurate intracerebral recording of electrical activity, it is imperative that the planned trajectory of the depth electrodes correlates with their actual trajectory, and therefore, there must be absolutely no patient movement during the procedure.

ECoG is performed to confirm positioning and ensure that all electrodes are functional; certain anesthetic drugs could easily affect these signals. Optimal ECoG readings are obtained at a stable, low-level anesthetic depth, and thus, there is a risk of patient recall [8]. It is reasonable to use propofol to facilitate induction, especially since its effects will be insignificant by the time ECoG is performed. Propofol in sedative doses have a minimal effect on ECoG recordings, but larger doses, which may be required during seizure activity, may obscure interictal discharges during ECoG [9].

Dexmedetomidine and low-dose boluses or continuous intravenous infusions of opioids have minimal effect on ECoG signals [8]. Based on our experience, a continuous remifentanil infusion along with boluses of fentanyl for postoperative pain control appears to be the ideal opioid combination for these types of cases, especially since local anesthetic is injected by the neurosurgeon at the burr hole incision site. Remifentanil is easily titratable and can be administered at higher infusion rates that decrease the chance of patient movement when paralytic cannot be used (i.e., when motor evoked potentials are being monitored). Additionally, using remifentanil during the operation will not hinder emergence, the need for an awake extubation, or an immediate postoperative neuro exam, due to its context-sensitive half time. We recognize that in our case series, one patient received a sufentanil infusion, while nine patients were maintained with fentanyl and/or morphine boluses. This occurred due to recent remifentanil drug shortages during the times of those patient’s surgeries, so alternative opioid management techniques were implemented.

Inhaled anesthetic agents have been shown to suppress spontaneous interictal spikes with background ECoG at 1 MAC; lower concentrations of inhaled anesthetics are used to minimize these suppressive effects. Sevoflurane and enflurane enhance nonspecific spike activity, while isoflurane and desflurane have minimal effects [8,10]. Both desflurane and isoflurane show no activation of spontaneous interictal epileptiform activities or neuroexcitation [9,10]. Low concentrations of isoflurane or desflurane are recommended along with the use of a continuous opioid infusion during ECoG. No patients in our case series were maintained on nitrous oxide during non-ECoG portions of the operation. Nitrous oxide’s effect on ECoG is controversial, with some studies suggesting a synergistic effect with halogenated agents, causing signal suppression, while other studies state that there is no effect on ECoG with nitrous oxide concentrations as high as 70% [8,10,11]. Except during the brief interval when ECoG is being recorded, nitrous oxide is usually avoided due to the rare risk of pneumocephalus.

In all cases, at least two large-bore IV catheters were placed due to the patient’s arms being tucked and difficult to access during the procedure. Arterial lines were not routinely inserted because intracerebral hemorrhage and the need for transfusion are extremely rare in SEEG electrode implantation. In those with increased risk for intraoperative bleeding or complex medical history, an arterial line for invasive hemodynamic monitoring was considered. Of note, arterial lines were placed in initial cases at our institution, but invasive monitoring utilization has since decreased as we have gained more experience managing these cases. As far as blood pressure management, hypertension was avoided in the rare event of intracerebral hemorrhage from depth electrode placement. Conversely, hypotension was also avoided since decreased cerebral perfusion could have resulted in interference with neuromonitoring and depth ECoG. Only five patients required small doses of ephedrine for persistent hypotension during ECoG. One patient received a platelet transfusion at the request of the neurosurgeon based on the appearance of the surgical field.

There is a complication rate of 13% with subdural grid and strip electrode placement as compared to only 0.18% per electrode for robot-assisted SEEG placement [1,10,12]. Intracranial hematomas, diagnosed by CT, after robot-assisted procedures, are usually self-limited and do not require surgical intervention [13]. It is important to note that there has been one isolated report of SEEG electrode breakage during a seizure [13]. At our institution, there have been no reported or observed complications with placement or removal of SEEG electrodes. A postoperative CT should be considered and decided on a case-by-case basis at experienced institutions. If there are any intraoperative complications or acute changes on neurological exam in the postoperative period, a CT might be necessary. Neurosurgeons who are just implementing ROSA^®^ at their institution might want to consider obtaining a postoperative CT routinely until their program is more established. While all patients in this case series had iMRI or postoperative CT to rule out an intracranial bleed, we no longer obtain postoperative imaging unless clinically justified.

ROSA^®^ depth electrode placement was used for stereotactic implantation of a Visualase^®^ laser probe in five of our patients. Visualase^®^ uses real-time iMRI thermography for seizure foci ablation, adding to the total operative time. The number of electrodes placed in each patient varies, also influencing surgical duration. This might explain the longer operative time at our institution compared to reported mean operative time of 107 min [1]. As with most emerging technology, there is a learning curve, and intraoperative efficiency continues to improve.

Eleven out of 19 patients have proceeded with resective or ablative surgery. Current literature describes a 55.5% seizure-free outcome with robot-assisted SEEG procedures following resection in the pediatric population [13]. Of our 11 patients, following resection or ablative surgery, 4 (36%) are seizure-free, 4 (36%) have a decrease in seizure frequency, and 3 (27%) report the quality and frequency of their seizures as unchanged. Five patients did not require a resective or ablative surgery after SEEG implantation because no focally localizable seizures were identified, or subsequent surgery was not advised due to seizure foci location. Using ROSA^®^ spared these patients from an unnecessary craniotomy that would have occurred with subdural grid or strip electrode placement, which are associated with higher risk for blood loss, more postoperatively pain, and longer operative times. Even with SEEG implantation, seizures can be extremely difficult to localize in the pediatric population; thus, two patients required subsequent strip electrode placements to further localize seizure foci. In addition, ROSA^®^ has aided in localizing seizure foci allowing for subsequent placement of the NeuroPace^®^ (NeuroPace Inc., Mountain View, CA, USA) responsive neurostimulation (RNS) brain stimulator to aid in drug-resistant epilepsy at our institution. Limitations of this retrospective case series include a small sample size and a short follow-up interval for the more recent patients, restricting long-term outcome assessment. All patients continue to have follow-up with the neurosurgeon and neurologist.

## 5. Conclusions

ROSA^®^ allows for a safe, efficient, minimally invasive, and highly accurate image-guided approach to depth electrode placement. Image guidance along with the stability and precision of the robotic arm, offers notable advantages over traditional approaches. Future studies are needed to determine whether adopting this new technology could reduce perioperative complications and improve patient outcomes and disease severity. As more pediatric institutions implement ROSA^®^, anesthetic management protocols should be considered.

## Figures and Tables

**Table 1:** Demographics, intraoperative management, and outcomes.

Characteristic/Outcome	N (%)	Mean (SD)	Median (Range)
**Age** (years)	-	13 (5)	14 (2–21)
**Weight** (kg)	-	52 (21)	54 (14–102)
**Sex**			
male	17 (74)	-	-
female	6 (26)	-	-
**Induction of anesthesia**			
Inhalational (8% sevoflurane + 70% nitrous oxide)	18 (78)	-	-
Intravenous (2% lidocaine 1 mg/kg + propofol 2–3 mg/kg)	5 (22)	-	-
**Vascular access**			
2 peripheral IV catheters	23 (100)	-	-
radial arterial line	7 (30)	-	-
**Maintenance of anesthesia**			
sevoflurane (MAC 0.5)	20 (87)	-	-
isoflurane (MAC 0.5)	3 (13)	-	-
remifentanil (0.1–1 µg/kg/min)	13 (57)	-	-
sufentanil (0.1–0.25 µg/kg/h)	1 (4)	-	-
no continuous opioid infusion	9 (39)	-	-
fentanyl (µg/kg)	22 (96)	2.2 (1.4)	1.9 (0–4.4)
morphine (mg/kg)	7 (30)	0.02 (0.04)	0 (0–0.14)
rocuronium used	18 (78)	-	-
**Vasopressors**			
none	18 (78)	-	-
ephedrine (0.04–0.15 mg/kg)	5 (22)	-	-
**Fluid administration**			
crystalloid (mL/kg)	23 (100)	25 (10)	27 (8–46)
5% albumin (mL/kg)	7 (30)	2 (4)	0 (0–11)
blood product transfusion (mL/kg)	1 (4)	0.2 (0.9)	0 (0–4.4)
**Urine output (mL/kg)**	23 (100)	4 (3)	3 (0.4–11.5)
**Extubated at end of surgery**			
no	0 (0)	-	-
yes	23 (100)	-	-
**Operative time** (minutes)	-	148 (60)	129 (67–252)
**Number of SEEG electrodes placed**	148	6 (3)	6 (2–13)
**Complications**			
intraoperative	0 (0)	-	-
postoperative	0 (0)	-	-
**Postoperative CT**	18 (78)	-	-
**PICU length of stay** (days)	-	1 (0)	1 (1–1)
**Total hospital length of stay** (days)	-	9 (6)	8 (2–29)

IV: intravenous; MAC: minimum alveolar concentration; SEEG: stereoelectroencephalography; CT: computed tomography; PICU: pediatric intensive care unit.

**Table 2:** Characteristics and surgical outcomes for 23 ROSA^®^ SEEG procedures in 19 pediatric patients.

Patient Number	Age (Years)	Weight (kg)	Sex	Diagnosis	Prior Neurosurgical Intervention	Number of Electrodes Placed	Operative Time (Minutes)	Hospital [PICU] Length of Stay (Days)	Postoperative Imaging	Subsequent Surgery Based on SEEG Results	Seizure Outcome after Resective/Ablative Surgery
1	17	102	M	epilepsy	none	4	116	8 [1]	CT	none	N/A
2	14	53	M	epilepsy, *DEPDC5/HNRNHP1* genes	none	10	245	22 [1]	CT	none	N/A
3	8	35	M	epilepsy	anaplastic ependymoma resection	9	104	11 [1]	CT	seizure focus resection	seizure free
4	14	71	M	epilepsy	none	8	158	10 [1]	CT	NeuroPace, DBS pending	N/A
5	21	54	M	epilepsy	cortical dysplasia excision ×2	8	201	8 [1]	CT	Visualase with ClearPoint	seizure free
6	15	75	M	epilepsy	none	13	228	11 [1]	CT	none, NeuroPace pending	N/A
7	14	75	F	epilepsy	VNS, brain biopsy	9	136	13 [1]	CT	NeuroPace, DBS	↓ seizure frequency
8	6	23	M	tuberous sclerosis, epilepsy	tuber resection	8	99	8 [1]	CT	Visualase	unchanged
6						3 *	252	2 [1]	iMRI	none	
23											
9	17	55	M	epilepsy	frontal lobe seizure focus resection	5	80	7 [1]	CT	Visulase	unchanged
17						2 *	152	2 [1]	iMRI	none	
55											
10	12	58	F	epilepsy	frontal cortical dysplasia excision ×3	4	67	11 [1]	CT	Visualase	↓ seizure frequency
12						2 *	209	2 [1]	iMRI	none	
58											
11	16	74	M	epilepsy	none	12	129	12 [1]	CT	grid placement, partial resection of temporal lobe, right amygdalohippocampectomy	↓ seizure frequency
12	12	52	F	Aicardi syndrome, epilepsy	none	2 *	186	2 [1]	iMRI	none	N/A
13	2	14	M	tuberous sclerosis, epilepsy	none	5	124	9 [1]	CT	resection of right inferiomedial frontal calcified tumor	unchanged
14	15	54	M	epilepsy	right frontal lobectomy	6	91	8 [1]	CT	Visualase	↓ seizure frequency
						2 *	216	2 [1]	iMRI	none	
15	20	56	M	epilepsy	none	5	71	12 [1]	CT	temporal lobectomy	seizure free
16	14	46	F	epilepsy	temporal lobectomy	4	100	14 [1]	CT	left temporal lobectomy, left hippocampectomy	seizure free
17	8	81	M	epilepsy	none	10	225	6 [1]	CT	grid placement and removal, NeuroPace	N/A
18	7	23	M	oligodendroglioma	tumor resection ×3	7	89	29 [1]	CT	none	N/A
19	12	55	F	epilepsy	seizure focus resection	10	124	8 [1]	CT	none	N/A

* ROSA^®^ electrode placement used for Visualase^®^; VNS = vagal nerve stimulator; DBS = deep brain stimulation; iMRI = intraoperative magnetic resonance imaging. ↓ = decrease.
